# Index Blood Tests and National Early Warning Scores within 24 Hours of Emergency Admission Can Predict the Risk of In-Hospital Mortality: A Model Development and Validation Study

**DOI:** 10.1371/journal.pone.0064340

**Published:** 2013-05-29

**Authors:** Mohammed A. Mohammed, Gavin Rudge, Duncan Watson, Gordon Wood, Gary B. Smith, David R. Prytherch, Alan Girling, Andrew Stevens

**Affiliations:** 1 Primary Care Clinical Sciences, University of Birmingham, Edgbaston, Birmingham, United Kingdom; 2 Unit of Public Health, Epidemiology and Biostatistics, University of Birmingham, Edgbaston, Birmingham, United Kingdom; 3 University Hospital Coventry and Warwickshire, Coventry, United Kingdom; 4 George Eliot Hospital, Nuneaton, Warwickshire, United Kingdom; 5 Centre of Postgraduate Medical Research and Education, School of Health and Social Care, Bournemouth University, Royal London House, Bournemouth, Dorset, United Kingdom; 6 Portsmouth Hospitals NHS Trust, Portsmouth, United Kingdom; 7 Centre for Healthcare Modelling, School of Computing, University of Portsmouth, Portsmouth, United Kingdom; The University of Tennessee Health Science Center, United States of America

## Abstract

**Background:**

We explored the use of routine blood tests and national early warning scores (NEWS) reported within ±24 hours of admission to predict in-hospital mortality in emergency admissions, using empirical decision Tree models because they are intuitive and may ultimately be used to support clinical decision making.

**Methodology:**

A retrospective analysis of adult emergency admissions to a large acute hospital during April 2009 to March 2010 in the West Midlands, England, with a full set of index blood tests results (albumin, creatinine, haemoglobin, potassium, sodium, urea, white cell count and an index NEWS undertaken within ±24 hours of admission). We developed a Tree model by randomly splitting the admissions into a training (50%) and validation dataset (50%) and assessed its accuracy using the concordance (*c*-) statistic. Emergency admissions (about 30%) did not have a full set of index blood tests and/or NEWS and so were not included in our analysis.

**Results:**

There were 23248 emergency admissions with a full set of blood tests and NEWS with an in-hospital mortality of 5.69%. The Tree model identified age, NEWS, albumin, sodium, white cell count and urea as significant (p<0.001) predictors of death, which described 17 homogeneous subgroups of admissions with mortality ranging from 0.2% to 60%. The *c-*statistic for the training model was 0.864 (95%CI 0.852 to 0.87) and when applied to the testing data set this was 0.853 (95%CI 0.840 to 0.866).

**Conclusions:**

An easy to interpret validated risk adjustment Tree model using blood test and NEWS taken within ±24 hours of admission provides good discrimination and offers a novel approach to risk adjustment which may potentially support clinical decision making. Given the nature of the clinical data, the results are likely to be generalisable but further research is required to investigate this promising approach.

## Introduction

There is considerable interest in developing statistical models which can adjust for patient case-mix and predict the risk of death in hospital. Whilst there are several different models in widespread use [1], [2], [3], [4], [5], [6], [7] a common feature of existing approaches is that they generally rely on routinely collected clinically coded administrative databases [8]. Whilst such data are readily obtained [9] it is important to recognise that clinical coding is not an integral part of the clinical decision making process and this has serious implications (which ultimately can undermine risk adjustment schemes), viz:- (1) Differences in the clinical coding processes at different hospitals (or even amongst coders in the same hospital) can produce materially different primary and secondary diagnoses codes for the same patient episode. (2) The era of case-mix adjusted hospital mortality ratios has seen changes, some of which amount to gaming, in the clinical coding process usually aimed at reducing the headline mortality ratio, thereby giving the possibly misleading impression that mortality has fallen because of quality of care [10]. It is therefore unsurprising to learn that there is evidence that hospital mortality ratios may sometimes reflect differences in clinical coding practices as opposed to genuine differences in quality of care [11], [12], [13]. (3) The clinically coded data frame is completed after the patient has been discharged and so restricts case-mix adjustment models that rely on coded data to retrospective use with no prospect of real-time decision support for clinical decision making.

Given these limitations we sought risk adjustment covariates from other routinely collected data sources which could be used to predict the risk of patients dying in hospital. Notwithstanding the use of such data to produce hospital mortality ratios, our primary focus is on developing tools which may ultimately support clinical decision making in real time. Candidate covariates, therefore, need to be routinely available on, or near, admission, be integral to the clinical decision making process and be measured with high quality without being susceptible to gaming. As most deaths in hospital occur in patients admitted as emergencies, we focused on these and noted that very early into their care, almost all of these patients will have a routine blood test and, at least in the National Health Service (NHS), an early warning score (EWS) derived from a range of vital signs (e.g. respiration rate, blood pressure, heart rate, temperature) measurements [14]. These data are not subject to many of the shortcomings of administrative clinically coded data. Laboratory tests are subject to strict quality control, and their results are clinically meaningful, have face-validity and are collected as part of the process of care. Similarly, EWS values generated from physiological measurements are part of the care process and are unlikely to be intentionally altered, although their accuracy depends upon correct equipment calibration, measurement technique and the arithmetical skills of staff, although it has been shown that electronic aids can improve accuracy [15], [16], [17].

Since our focus is on supporting clinical decision making, we used Classification and Regression Trees, (CART), which is a statistical data mining technique for constructing empirical decision Trees by recursively splitting or partitioning patients into homogenous subgroups [18]. Tree models have been used to support medical decision making [19], [20], [21]_._Although their use is still somewhat novel, Tree models, unlike regression models, are intuitive to interpret because they have a simple flowchart type presentation which starts by identifying the most important predictor variables, naturally incorporate interaction effects, identify cut-offs for continuous covariates, are distribution free and can handle non-linear relationships. Some of these characteristics reflect human decision making processes.

We therefore sought to determine if the combination of routine blood test results and electronically collected EWS values could be used in a Tree model to predict in-hospital mortality with a reasonable degree of accuracy.

## Methods

### Setting and Data

Our cohort of emergency admissions is from University Hospitals Coventry and Warwickshire NHS Trust, one of the largest acute teaching hospitals in the UK with 1250 beds. All spells following emergency admission within the period April 2009 to March 2010 were included. For each admission we obtained the following: patient’s age, gender, admission date/time, discharge date/time and discharge status (alive/dead). The following were excluded - patients aged less than 16 years of age, admissions to the maternity unit or any admissions with missing or invalid data. Using a pseudonymised, unique patient identifier, we obtained linked admission spells with the database of EWS values collected from patients’ vital signs using commercially available, personal digital assistants running specifically designed software (VitalPAC™, The Learning Clinic Ltd, London) [22]. VitalPAC™ has a series of robust real-time data validation checks to ensure that valid physiological measurements are entered at the point of care and accurate EWS are automatically generated [22]. The index EWS was defined as the first recorded value within a ±24 hour window either side of the admission date/time. We converted the local EWS into the recently published National EWS (NEWS) [23] for the NHS to aid generalisability. This conversion from EWS to NEWS was straightforward because EWS and NEWS have the same seven underlying physiological variables (respiration rate, oxygen saturations, any supplemental oxygen (yes/no), temperature, systolic blood pressure, heart rate and level of consciousness (Alert, Voice, Pain, Unresponsive). The accuracy of this conversion was assured by using the actual physiological measurements that was entered in VitalPAC™ and mapping these onto the NEWS scoring system [23].

These emergency admissions data were linked using unique (but de-identified) patient identifiers to the hospital laboratory computer system to determine the first blood test reported within a ±24 hour window (either side of the admission date/time). We included tests and EWS up to 24 hours before the admission date because it is not unusual for patients to have these available in the Accident and Emergency (A&E) department just before being formally admitted to the hospital. Blood tests outside this ±24 hour window were not regarded as index blood tests and were excluded. Emergency admissions without a full set of data were excluded from this analysis.

We considered the following commonly undertaken seven blood tests:- albumin (g/L), creatinine (µmol/L), haemoglobin (g/dL), potassium (mmol/L), sodium (mmol/L), urea (mmol/L) and white cell count (WCC) (10^9^ cells/L) because they are routinely undertaken. Like most hospitals, the blood tests results in UHCW are subject to day-to-day internal and external quality assurance [24]. The NEWS ranged from 0 (indicating the lowest severity of illness) to 19 (the maximum NEWS value possible is 20), although we capped this at 8, because only 346 (1.49%) admissions had higher scores.

### Statistical Analyses

We began by undertaking exploratory analysis of the NEWS and the blood test results. To avoid undue influence of extreme outliers to the modelling process, very high (> = 99.9% centile) or very low (< = 1% centile), blood test results were truncated to the limit of the inequality where necessary. We produced scatter plots showing the relationship between mortality and NEWS and the blood test results (grouped into sextiles).

We modelled the risk of death using Classification and Regression Trees. Our Tree modelling strategy involved the following covariates – age, gender, albumin, creatinine, haemoglobin, potassium, sodium, urea, WCC and NEWS. When first developed, CARTs could lead to quite large Tree models, but recent work has incorporated *p*-value based Tree modelling, known as conditional Trees, which yield smaller Tree models whilst simultaneously controlling for multiple testing, (Bonferroni adjustment, based on p≤0.001). They are available in the *Party* Package [25] in *R*. The Tree models produce a flowchart type of output which is intuitive. The Tree models have nodes (shown as ovals) and branches (shown as lines) and end up locating homogeneous subgroups in terminal nodes which are represented by a rectangular box at the bottom of the Tree with the sample size indicated by n =  and the risk of death as y = . Tree models can also be summarised in nested tabular format to which we added 95% binomial confidence intervals using the *exactci* package [26] in *R* [27]. All analyses were undertaken in *R*.

We randomly divided our dataset into a training set (n = 11624) and a testing set (n = 11624) for model validation [28]. In assessing the Tree model we used the concordance measure of discrimination – the *c-*statistic. For a binary outcome, the *c-*statistic is the area under the Receiver Operating Characteristics, (ROC) [29] curve. The ROC curve is a plot of the sensitivity, (true positive rate), versus 1-specificity, (false positive rate), for consecutive predicted risks. The area under the ROC curve is interpreted as the probability that a deceased patient has a higher risk of death than a randomly chosen non-deceased patient. A *c-*statistic of 0.5 is no better than tossing a coin, whilst a perfect model has a *c-*statistic of 1. The higher the *c-*statistic the better the model. In general, values less than 0.7 are considered to show poor discrimination, values of 0.7 to 0.8 can be described as reasonable, and values above 0.8 suggest good discrimination. The 95% confidence interval for the *c-*statistic was derived using DeLong’s method as implemented in the *pROC* library [30] in *R* [31].

### Ethical Approval

The lead author (MAM) sought advice from chair of the Birmingham research ethics committee and was advised that formal ethical approval was not necessary because this is an audit/service evaluation of existing hospital data. However, a co-author (DP) has obtained ethical approval from the Isle of Wight, Portsmouth & South East Hampshire Research Ethics Committee (Reference No: 08/02/1394) for desktop analysis of routinely collected NHS data. All data was de-identified prior to analysis. The datasets used in our study are routinely collected as part of the process of care whilst patients stay in hospital and therefore do not require specific consent.

## Results

**Figure 1:**
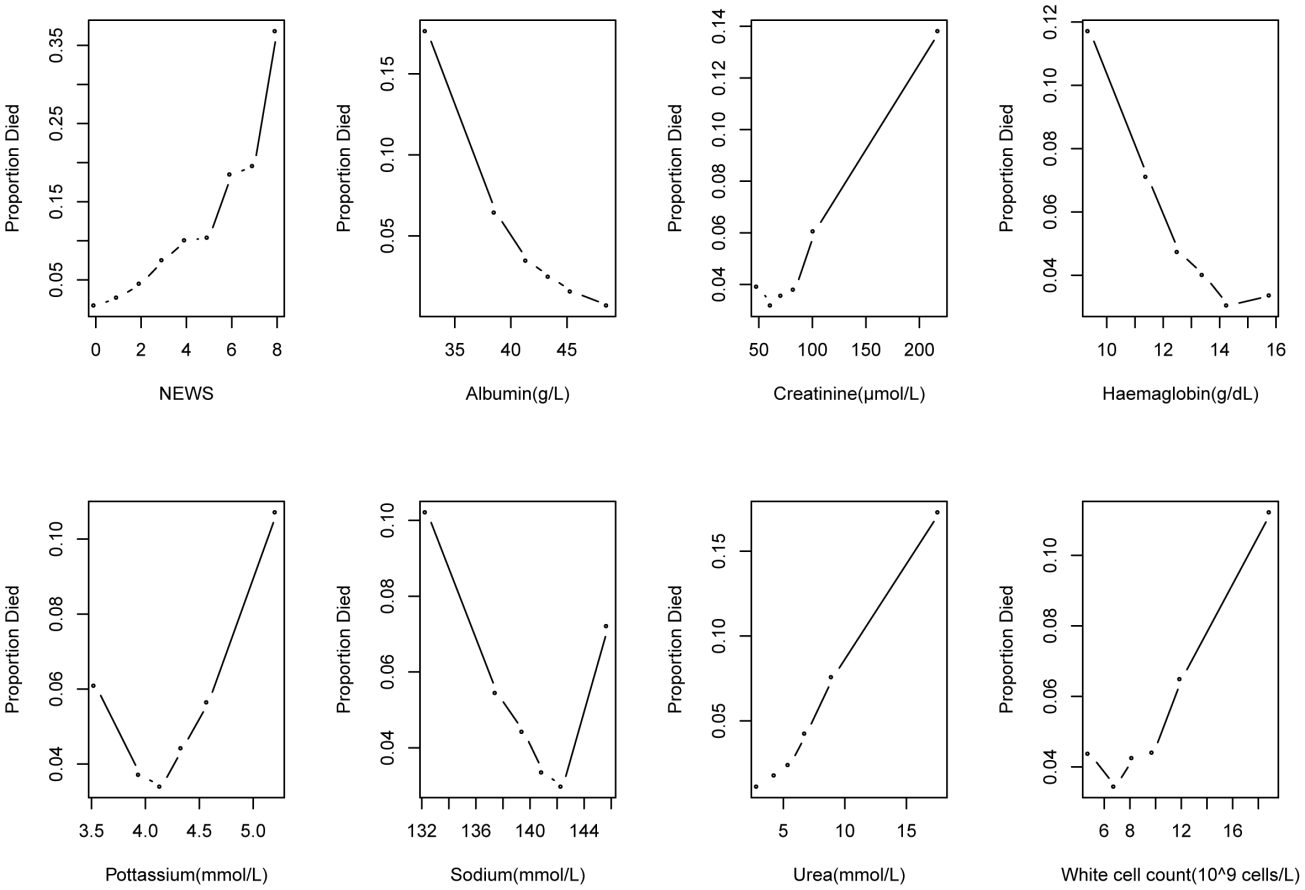
Scatter plots showing observed risk of death with NEWS and seven blood tests results.

There were 23248 emergency admissions from April 2009 to March 2010, with a mean age of 61.54 years (SD 21.59), a female to male ratio of 1.09 and accompanying hospital mortality of 5.69% (1323/23248). Table 1 shows the mean and standard deviationsfor the individual blood test results and index NEWS values in admissions that were discharged alive and dead. In general, patients who died were older, had higher index NEWS, lower albumin, higher creatinine, lower haemoglobin, higher potassium, higher urea and higher WCC. Little difference was seen in the mean sodium values. Figure 1 shows the relationship between these variables and mortality. All variables showed a mostly non-linear relationship with mortality.

**Table 1:** Mean and (standard deviation) for the continuous covariates in the Tree model.

Variable	Alive (n = 21925)	Died (n = 1323)	All (n = 23248)
Age	60.52 (21.59)	78.46 (12.70)	61.54 (21.59)
NEWS	1.79 (1.85)	3.98 (2.61)	1.95 (2.10)
Albumin	41.71 (5.20)	35.66 (5.67)	41.37 (5.41)
Creatinine	95.56 (75.53)	136.11 (106.28)	97.87 (78.17)
Haemoglobin	12.87 (2.12)	11.73 (2.32)	12.81 (2.15)
Potassium	4.27 (0.57)	4.46 (0.84)	4.28 (0.59)
Sodium	139.40 (4.60)	138.10 (7.04)	139.33 (4.78)
Urea	7.38 (5.15)	13.53 (8.20)	7.73 (5.56)
White cell count	9.97 (5.82)	13.16 (10.77)	10.16 (6.25)

**Figure 2:**
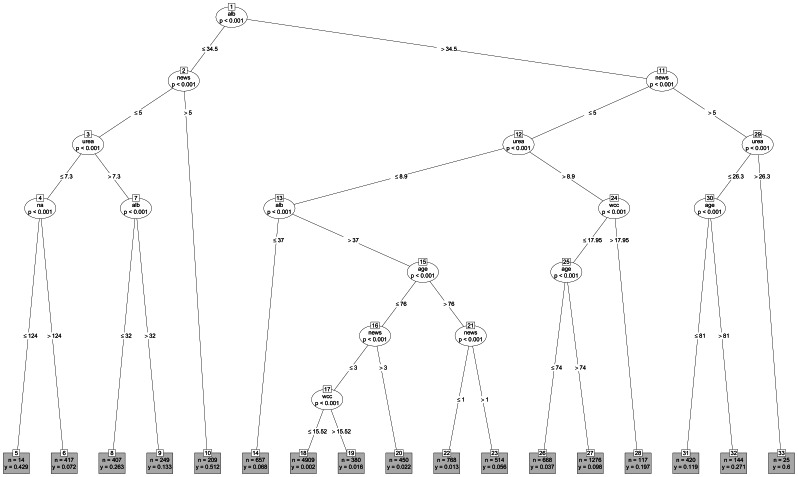
The Tree model.

We developed a Tree model (see figure 2) with age, NEWS and the seven blood test results. The *c-*statistic for the training model was 0.864 (95%CI 0.852 to 0.876) and when applied to the testing data set this was 0.853 (95%CI 0.840 to 0.866).

The Tree identified age, NEWS, albumin, WCC and urea as significant predictors of death. The appearance of some of these covariates (eg age, albumin) in more than one branch of the Tree model reflects the presence of significant interaction effects, wherein the relationship between a given covariate and risk of death changes depending on its context with respect to other covariates in the Tree. The Tree model involved 33 nodes of which 17 were terminal nodes (grey rectangles at bottom of Tree). For convenience, the 17 terminal nodes (ie the 17 homogenous subgroups) are summarised in table 2 along with 95% confidence intervals. The highest risk was seen in terminal node 33 which involved 25 emergency admissions with a 60% risk of death. These admissions were characterised by albumin >34.5, NEWS >5 and urea>26.3. The lowest risk of death (0.2%) was seen in terminal node 18 which had the largest sample size (n = 4909) and characterised emergency admissions with age ≤76, NEWS ≤3, albumin >37, urea ≤8.9 and WCC ≤15.52.

**Table 2:** Shows the characteristics of admission in the seventeen terminal nodes (in descending risk) of the Tree model.

Characteristics from Tree model	Terminal Node	Sample size	Mortality Risk (95% Confidence Interval)
(1) alb >34.5; (11) news >5; (29) urea >26.3; (33)*	33	25	0.600(0.387 to 0.789)
(1) alb ≤34.5; (2) news >5; (10)*	10	209	0.512(0.442 to 0.582)
(1) alb ≤34.5; (2) news ≤5; (3) urea ≤7.3; (4); na ≤124; (5)*	5	14	0.429(0.177 to 0.711)
(1) alb >34.5; (11) news >5; (29) urea ≤26.3; (30); age >81; (32)*	32	144	0.271(0.200 to 0.351)
(1) alb ≤34.5; (2) news ≤5; (3) urea >7.3; (7); alb ≤32; (8)*	8	407	0.263(0.221 to 0.309)
(1) alb >34.5; (11) news ≤5; (12) urea >8.9; (24); WCC >17.95; (28)*	28	117	0.197(0.129 to 0.280)
(1) alb ≤34.5; (2) news ≤5; (3) urea >7.3; (7); alb >32; (9)*	9	249	0.133(0.093 to 0.181)
(1) alb >34.5; (11) news >5; (29) urea ≤26.3; 30); age ≤81; (31)*	31	420	0.119(0.090 to 0.154)
(1) alb >34.5; (11) news ≤5; (12) urea >8.9; (24); WCC ≤17.95; (25) age ≤74; (26)*	27	1276	0.098(0.082 to 0.116)
(1) alb ≤34.5; (2) news ≤5; (3) urea ≤7.3; (4); na >124; (6)*	6	417	0.072(0.049 to 0.101)
(1) alb >34.5; (11) news ≤5; (12) urea ≤8.9; (13); alb ≤37; (14)*	14	657	0.068(0.050 to 0.091)
(1) alb >34.5; (11) news ≤5; (12) urea ≤8.9; (13); alb >37; (15) age >76; (21) news ≤1; (22)*	23	514	0.056(0.038 to 0.080)
(1) alb >34.5; (11) news ≤5; (12) urea ≤8.9; (13); alb >37; (15) age >76; (21) news >1; (23)*	26	668	0.037(0.024 to 0.055)
(1) alb >34.5; (11) news ≤5; (12) urea ≤8.9; (13); alb >37; (15) age ≤76; (16) news >3; (20)*	20	450	0.022(0.011 to 0.040)
(1) alb >34.5; (11) news ≤5; (12) urea ≤8.9; (13); alb >37; (15) age ≤76; (16) news ≤3; (17) WCC >15.52; (19)*	19	380	0.016(0.006 to 0.034)
(1) alb >34.5; (11) news ≤5; (12) urea ≤8.9; (13); alb >37; (15) age ≤76; (16) news >3; (20)*	22	768	0.013(0.006 to 0.024)
(1) alb >34.5; (11) news ≤5; (12) urea ≤8.9; (13); alb >37; (15) age ≤76; (16) news ≤3; (17) WCC ≤15.52; (18)*	18	4909	0.002(0.001 to 0.003)

Nb: to be read in conjunction with the Tree model.

(Number) is node number.

* indicates a terminal node. Nodes are separated by;.

alb = albumin, news = NEWS, na = Sodium, WCC = white cell count.

## Discussion

We have used a simple set of clinical variables based on the index routine blood test results and NEWS to develop a validated risk adjustment model for in-hospital mortality following emergency admission. Unlike case-mix adjustment schemes that rely on administrative datasets, our approach has employed covariates that are routinely measured, routinely quality assured/audited, are an integral part of the process of care, are not susceptible to gaming, have clinical face-validity and are an established clinical currency within and between hospitals. These features of the covariate set are important because they help to protect against two major issues in case-mix adjustment – the constant risk fallacy [32] (where statistical adjustment increases bias) and the case-mix adjustment fallacy [33] (where differences in adjusted mortality rates does not reflect quality of care). The constant risk fallacy can occur, for example, when two identical patients have differing covariates (eg two identical unconscious stroke patients, but one gets a primary diagnosis label of syncope and the other as stroke; risk adjustment based on these diagnostic labels may now increase, not decrease, bias). In our approach it is not usually possible for two identical patients to have different blood test results even if different hospitals use different reference ranges. Our approach is immune to this because we use the actual values and ignore the reference ranges. It is however possible, especially using pen and paper methods, that two identical patients may have different NEWS, although the use of electronic aids appears to have addressed such measurement problems [17], [34]. The case-mix adjustment fallacy operates when it is presumed that the residual unexplained variation in risk-adjusted mortality reflects quality of care. In risk-adjustment schemes that use administrative data sets, difference in adjusted rates can often simply reflect differences in clinical coding practices as opposed to quality of care [11]. In our approach there is much less scope for identifying problems with the underlying data and hence more scope for identifying clinical issues and this is essential for any tool aimed at supporting the clinical decision making process.

Several previous studies have suggested the potential role of blood test results [35], [36], [37], [38], [39], [40], [41], [42] or patient physiology [43], [44] but few studies have combined these two data sources [45], [46], [47]. In our case, this was more feasible because UHCW uses an electronic system for EWS calculation [18]. In some respects the finding that a valid model can be obtained using the patient’s age, routine blood tests results and NEWS, is unsurprising because these variables have been an integral part of hospital medicine for decades, and as the scatter plots (figure 2) show, they have an empirical relationship with the risk of death. Although the Tree model did not identify all seven blood tests, it would be incorrect to infer that the blood tests (eg potassium, haemoglobin) that were not included are therefore of little clinical use. The usefulness or otherwise of a blood test is a much broader question involving differential diagnoses, whereas here we are concerned with developing a parsimonious model to predict mortality, with the potential for consideration as an index of quality of care/hospital performance.

The data used in our study comes from one of the largest acute hospitals in the NHS. These data form integral components of patient care and clinical decision making in a modern NHS hospital and suggest that our approach is transferable to other hospitals. Generalisability is further enhanced by our use of the recently introduced NEWS [19] for the National Health Service. However, since we analysed data from patients who have both a full set of index routine blood tests results and an index EWS within ±24 hours of admission, our findings are only valid for such emergency admissions (about 70%). We necessarily excluded a considerable proportion (about 30%) of emergency admissions (typically with a median hospital stay of zero days) because of missing data. Whilst these admissions merit further study (eg some may be too sick to undergo such assessments whilst others may be too well), it seems reasonable to presume that in general, they are not representative of those patients who go through a pathway of care which accrues an index blood test and an EWS.

Mandating that these emergency admissions should also have an index blood test and EWS is difficult to justify because a sophisticated mortality risk assessment tool is likely to be of minimal use in these patients. We also excluded elective admissions from our analysis because mortality is a rare event in the elective setting, although future studies could incorporate elective admissions, subject to the availability of the necessary data items. In addition, diagnoses and comorbidity labels are absent in the Tree model because they are not necessarily known or recorded (electronically) with a high degree of belief within ±24 hours of admission. Likewise, it is worth emphasising that administrative data sets also, in general, omit blood test results and EWS. O’Sullivan et al found that including comorbidity did not improve the accuracy of a laboratory data model to predict mortality in emergency admissions [48] suggesting that the omission of comorbidity in our approach may not be a major limitation.

The validated Tree model reported here has two potential uses. Retrospective use could take the form of clinical audit and review of deceased patients in a specific terminal node (eg node 18) with a very low risk of death (to check for suboptimal care) or with a high risk of death (eg node 33, to review the quality of end-of-life care perhaps). The Tree model can also be used to prospectively estimate the risk of death for patients within ±24 hours. This is akin to the practice of using risk models for managing critical care patients [49]. This estimated risk of death can be communicated to carers, patients and relatives to influence and inform the subsequent process of care. Since blood tests and EWS are in widespread use in hospital medicine, our approach could be developed (and properly evaluated) in any hospital. However, although our approach could also be used to develop case-mix adjusted hospital mortality ratios, we are cautious about this use because of unresolved methodological issues [2], [8], [12], [13], [14], [50]. Finally, it is worth emphasizing that ultimately the usefulness of a risk prediction model, irrespective of any impressive desktop statistical properties, is determined by the extent to which it enhances the delivery of high quality of care and reduces avoidable mortality [51], [52]. Our approach offers a promising start because it builds on established current clinical practice and has the potential to be integrated into the clinical decision making process but needs further research.
